# Supplementary material for: New hominin remains and revised context from the earliest Homo erectus locality in East Turkana, Kenya
Source: Nat Commun. 2021 Apr 13;12:1939. doi: 10.1038/s41467-021-22208-x (PMC8044126; doi:10.1038/s41467-021-22208-x)
Supplement: Supplementary file 1 — Supplementary Information [file 41467_2021_22208_MOESM1_ESM.pdf]

Supplementary Information for:

New hominin remains and revised context from the earliest *Homo erectus* locality in East Turkana, Kenya

Hammond et al.

This PDF file includes:

Supplementary Notes 1-2
Supplementary Figures 1-7
Supplementary Tables 1-2
Supplementary References 1-7

Supplementary Note 1: Sandstone mineralogy

The sandstones in the lower aggregate (upper Burgi Member) are heterolithic but have similar properties under thin section (Figure 4). They are moderately to well sorted sediments with variable mineralogical proportions (Figure 4). Oblate grains are typically aligned in the same orientation, suggesting a fabric. The sediments are mostly composed of grains and matrix with cement abundant in void spaces. These grains are usually subrounded with variable sphericity (largely as a function of the mineralogy). In addition, bioclasts are present in the form of diverse siliceous spicules from freshwater megascleres sponges. The most common grain mineralogy is in the form of both poly- and monocrystalline quartz and feldspars (microcline and plagioclase) present as well as other minerals including mica and possibly chlorite. Igneous rock fragments are infrequent and are usually very fine grained.

The coarse sandstones (KS) of the upper aggregate (KBS Member) shows consistent lithology in both texture and composition (Figure 4). KS is characterized by very poorly sorted and clast supported sediments that mimic the macroscopic outcrop. Under thin section, weak lamination is demonstrated by preferred grain orientation in the long axis, best illustrated by parallel arrangement of tabular grains. The grains comprise the majority of the sediment and loosely suspended in cement (calcite) although some matrix (<10%) can be observed. The grains are usually subspherical and subangular/angular in shape with although some less abundant mineralogy (micas) are tabular. The most common grain mineralogy is in the form of monocrystalline quartz with some feldspars (microcline and plagioclase) present as well as other minerals including mica and igneous rock fragments (which are abundant).

Supplementary Note 2: Hominin cranial vault fragments

There are 5 small fragments recovered at the KNM-ER 2598 locality that are likely to have originated from a hominin cranial vault (Supplementary Figure 5; KNM-ER 77066, KNM-ER 77067, KNM-ER 77068, KNM-ER 77069, KNM-ER 77070). Although they cannot be definitively identified to particular regions of the cranium, they are unlikely to be from the temporal or the occipital based on morphology. We recovered 4 additional fragments (not figured) that are also consistent with hominin cranial vault but which lacked any diagnostic morphology, and will not be considered here. All 5 of the fragments shown in Supplementary Figure 5 preserve unclosed suture borders as in KNM-ER 2598. The morphology of the cortical bone shows a similar texture as KNM-ER 2598 (Supplementary Figure 6), suggesting similar post-depositional processes. The new fragments are also consistent with the thickness of KNM-ER 2598 and most of these fragments also show the fairly divergent sutural limbs characteristic of KNM-ER 2598 and many *Homo erectus* crania [5]. However, none of the cranial fragments directly refit with KNM-ER 2598 nor can they be linked through surface morphology.

The fragments have proportionally thick diploe relative to cortical bone, as in other Plio-Pleistocene hominins (Supplementary Figure 4)[1,2]. A ternary plot demonstrates that hominin cranial bones, including the new fragments from the KNM-ER 2598 locality, have a larger proportion of diploe compared to outer and inner cortical bone thickness. The total thickness of

the fragments (6.7-9.3 mm) is consistent with the cranial vault thickness in most hominin taxa (e.g., *Australopithecus*, *Paranthropus*, *Homo*) except *Homo naledi*[1,3]. The data used to generate the plots in Supplementary Figure 4 are included in the Source Data file.

Supplementary Figure 1. KNM-ER 2598 reconstructed location. (a) A map of Kenya indicating the East Turkana region with a red box; (b) East Turkana shown with the boundaries of fossil collection areas[4,5] overlain on Google Earth imagery, with yellow box highlighting location of field activities and the yellow circle indicating the location where the sandstone cairn was found; (c) Area 13 is shown relative to Area 15; (d) Aerial photograph from the 1970s is compared to the same region as recently captured by satellite. The yellow arrows on the 1970s photograph indicate pinpricks used to mark fossil finds, with the pinprick documenting the KNM-ER 2598 location labelled. The KNM-ER 2598 location corresponds closely to the location in Area 13 where the cairn was found.

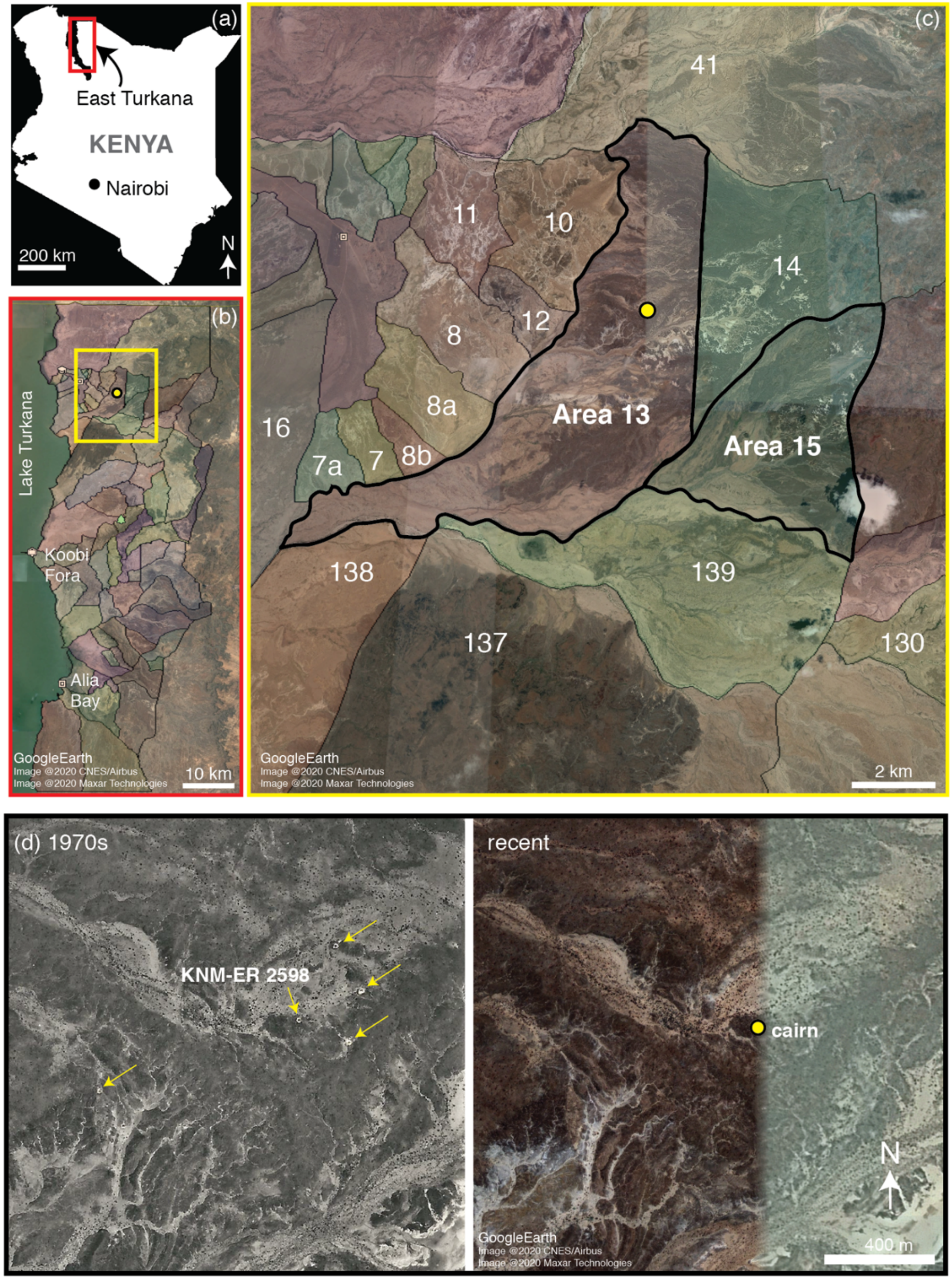

Supplementary Figure 2. Photographic evidence of 1970s collection activities within Area 13. (a) Photograph from the 1975 field season courtesy of T. D. White shows the distinctive large exposure of the KBS Tuff (KBT3 in Figures 2 and 3) which modern reconnaissance documents as being within boundaries of the modern-day Area 13. This large tuff is located at approximately 4.265190° N, 36.331777° E. (b) A close up view of the tuff in 1975 versus 2019, from different vantage points.

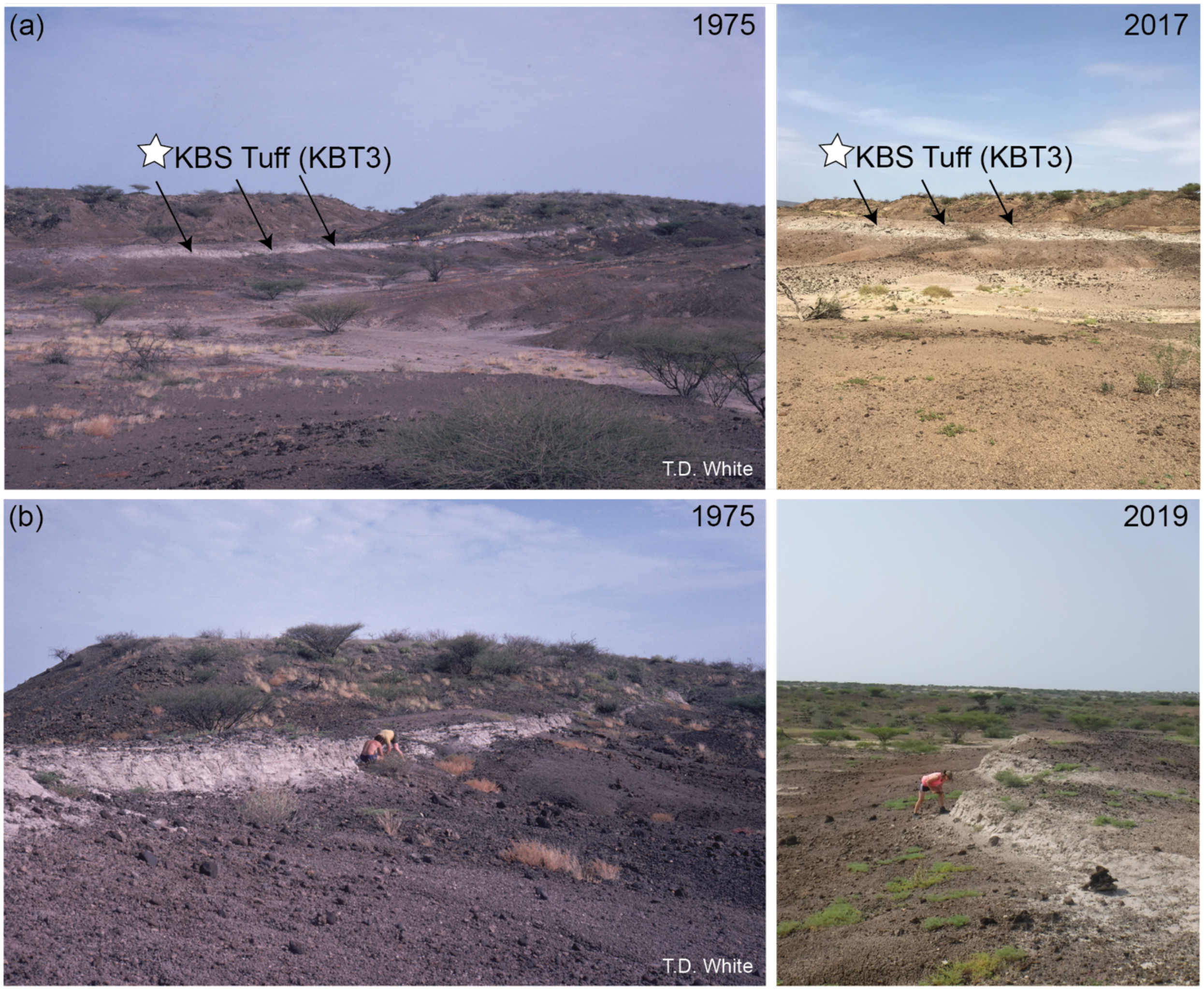

Supplementary Figure 3. The reconstructed KNM-ER 2598 location and fossil recovery in Area 13. (a-c) A pile of large sandstone rocks found at the reconstructed location of the KNM-ER 2598 site appears to be a collapsed human-made cairn, indicated in photos by a yellow dot. (d) Koobi Fora Field School students completing a surface crawl for fossil fragments just south of the cairn. (e) The entire Cluster-1 location (see Figure 3) is relatively flat with no higher elevation outcrops where fossils could erode from. (f) Fossiliferous Cluster-2 at the specific location where a *Ceratotherium* mandible (field identification 1511304/DP-ET-17-150) was recovered.

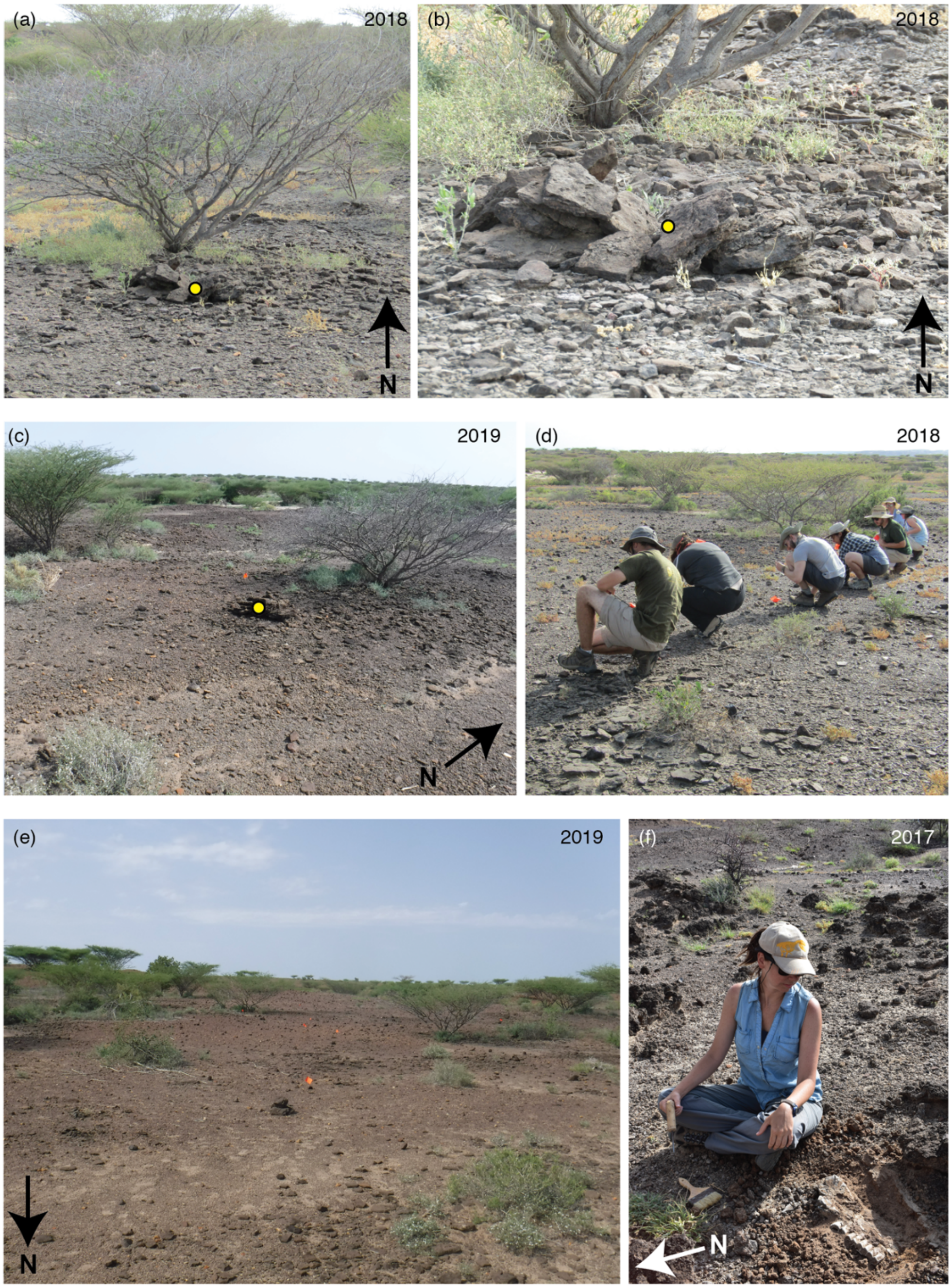

Supplementary Figure 4. Geological features. Inset map based on Figure 3 shows the location of photos. (a) A torrential drainage where upper Burgi (UB) outcrops are exposed below the KBS Tuff (KBT3). Note the relatively limited extent of the colluvial system with KS clasts that are washed away by the torrential drainage; (b) an occurrence with no apparent erosional boundary between the KBS Tuff (top) UB clays with carbonate concretions (base); (c) sub-horizontal outcrops of UB1 sandstones near KNM-ER 2598 locality, distant, on the other side of the torrential drainage the colluvial fans with KS clasts can be seen; (d) outcrops of laterally non-continuous (1) sandstones (UB2); (e) polymictic conglomerate (KS) with eroded clay clasts (2) and parallel stratification (3); (f) UB1 sandstone outcropping at KNM-ER 2598 locality with alternating horizontal (4) and trough cross-stratification (5).

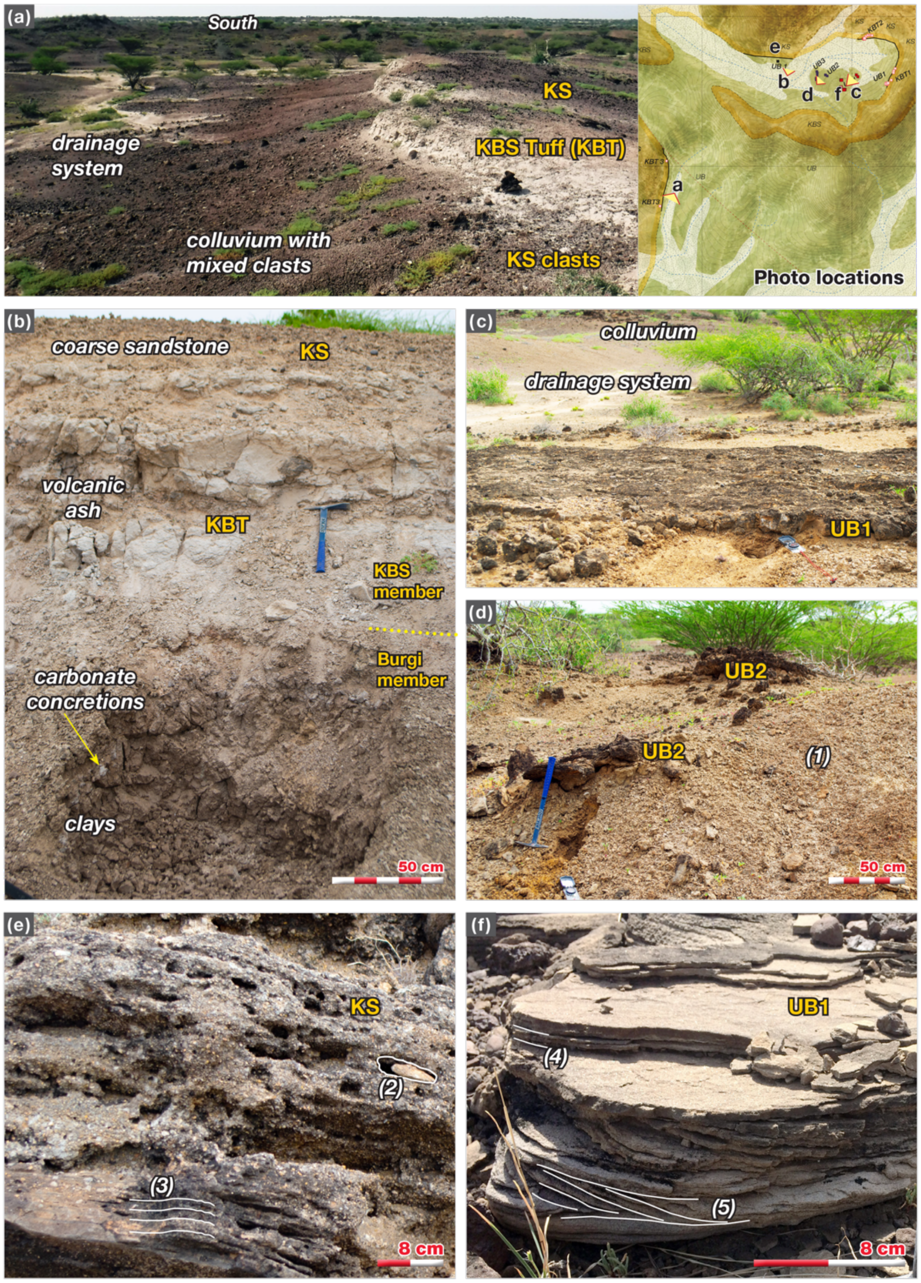

Supplementary Figure 5. Hominin cranial vault morphology and thickness. (a) Likely hominin cranial vault fragments. Unfused suture borders are oriented upwards. (b) A ternary plot for cranial bone composition, with the 95% confidence ellipse for hominin cranial bone indicated. (c-d) Frontal bone and parietal bone absolute thickness for Plio-Pleistocene hominins is shown as boxplots (center line=median; box limits=upper and lower quartiles; whiskers=1.5x interquartile range; points=outliers). The range of the new cranial fragments from the KNM-ER 2598 locality is indicated by the yellow box. Note that the dataset contains multiple values for some hominins, representing measurements taken at multiple locations on a single cranium, and so the range of the data shown for each species is the most meaningful information in the plot (i.e., boxplot quartiles affected by sampling multiple locations for each individual). Number of data points for panels c and d respectively, by species: *A. afarensis* (n=5, 54); *A. africanus* (18, 19); *P. boisei* (11, 22); early *Homo* (15, 52); *H. naledi* (1, 4); *H. floresiensis* (2, 2); *H. erectus* (11, 58); *H. erectus* Asia (64, 71); *H. erectus* Dmanisi (2, 0). Source data underlying figure are shown in Source Data File.

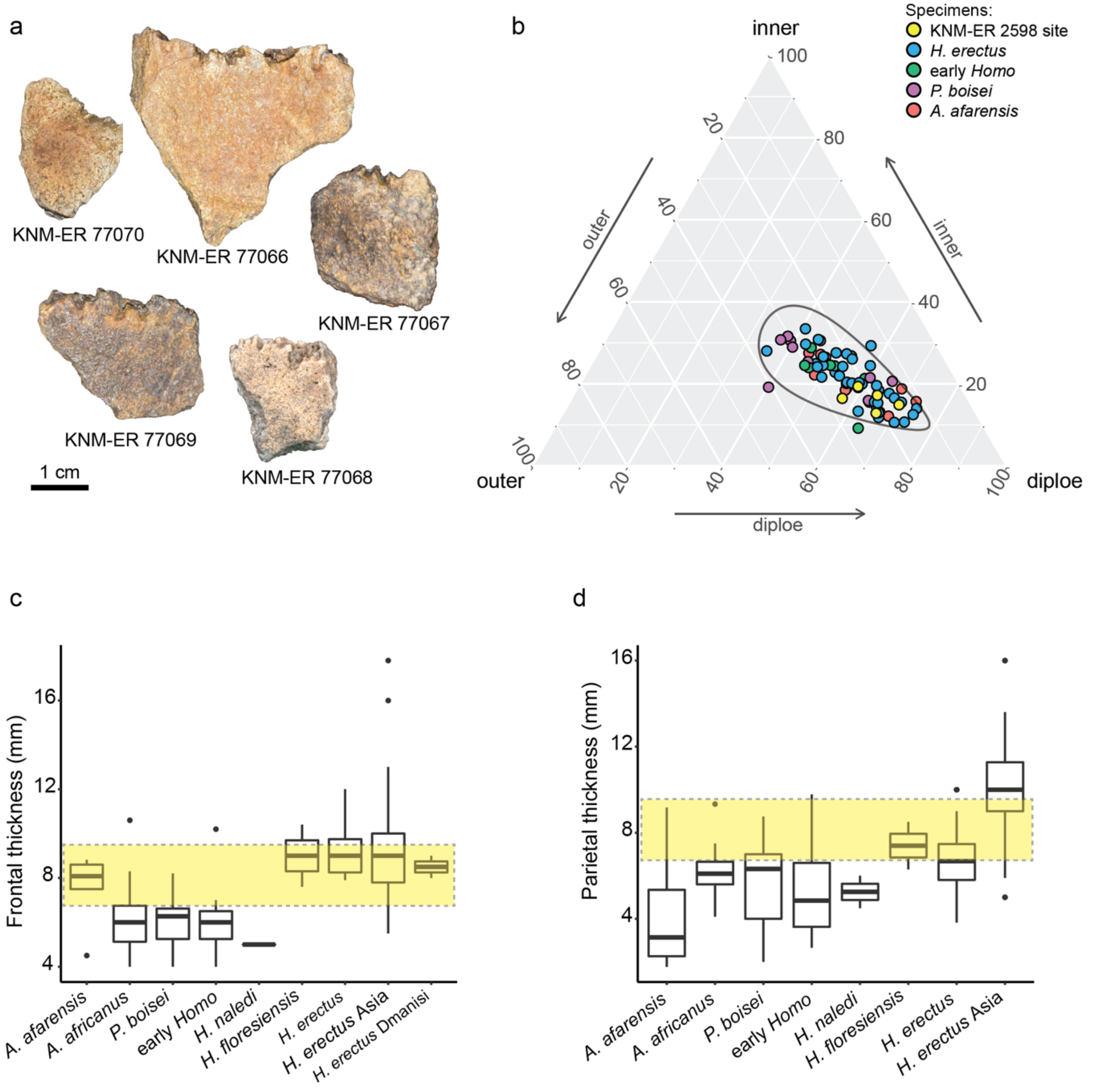

Supplementary Figure 6. Examples of cranial vault surface textures. The external and internal surfaces of (A) KNM-ER 2598, (B) fragment KNM-ER 77069, and (C) fragment KNM-ER 77066. KNM-ER 2598 and KNM-ER 77069 bear resemblance but cannot conclusively be associated to one another. Images were collected on a HIROX KH-7700 Digital Microscope at 160x magnification.

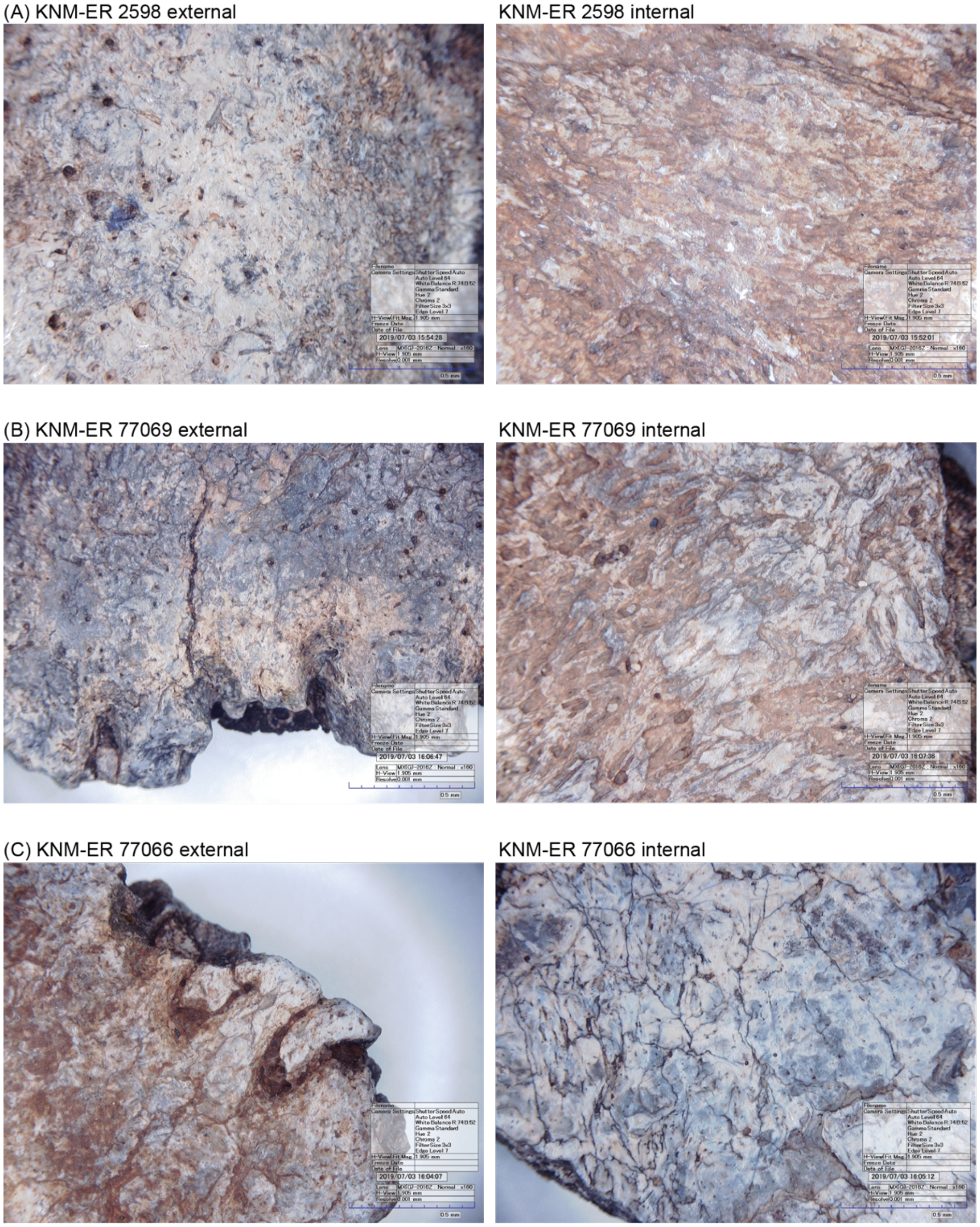

Supplementary Figure 7. Size of pelves from the upper Burgi Member of East Turkana, Kenya. Internal view above, external view below. KNM-ER 77072 is intermediate in size between KNM-ER 5881 and KNM-ER 3228, as evidenced by the size of the iliac tuberosity (IT) and length of the lower ilium (i.e., the region between the sacroiliac joint and acetabulum; LI). Note that the acetabulum is missing in KNM-ER 77072, requiring that the lower ilium would be longer than what is preserved. Yellow dashed line = the anterior border of the sacroiliac joint; red dashed line = rim of acetabulum (AR).

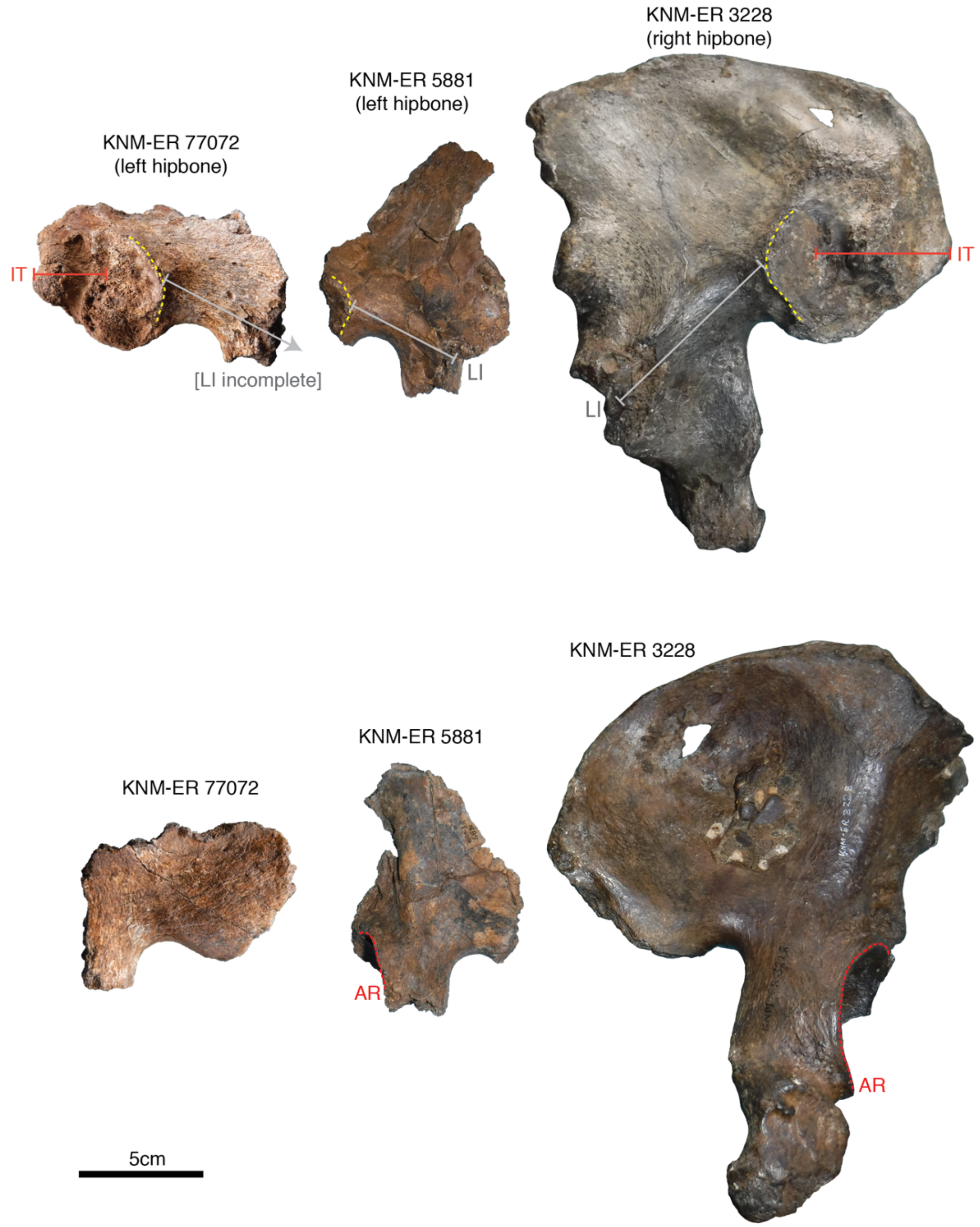

Supplementary Table 1. Isotope data[a]

Specimen	Family	Taxonomy	Element	$\delta^{13}C$	$\delta^{18}O^{a}$	%C4	± %C4
KNM-ER 77115	Bovidae	Alcelaphini	upper M3	-0.2	1.4	77	7
KNM-ER 77114 a	Bovidae	Alcelaphini	lower m1/m2	2.5	-0.7	94	3
KNM-ER 77114 b	Bovidae	Alcelaphini	upper M1/M2	1.8	0.3	90	4
KNM-ER 77120	Bovidae	Alcelaphini	molar fragment	1.7	3.6	89	4
KNM-ER 77118	Bovidae	Antilo/Aepycerotini	upper M3	1.5	1.3	88	4
KNM-ER 77113	Bovidae	Bovini	lower m3	1.4	0.1	87	4
KNM-ER 77103	Bovidae	Bovini	upper M1/M2	1.6	2.6	88	4
KNM-ER 77112	Bovidae	Reduncini	lower m3	1.3	-1.1	86	4
KNM-ER 77119	Elephantidae	*Elephas*	molar plate	1.2	-1.2	86	5
KNM-ER 77107	Equidae	*Equus*	lower molar	1.5	2.0	88	4
KNM-ER 77121	Equidae	*Equus*	lower m1/m2	1.7	2.9	89	4
KNM-ER 77108	Equidae	*Eurygnathohippus*	lower molar	0.4	-2.5	81	6
KNM-ER 77102	Hippopotamidae	*Hippopotamus*	upper M3	1.6	-3.5	88	4
field 1511304	Rhinoceratidae	*Ceratotherium*	lower m3 left	0.3	-1.7	80	6
KNM-ER 77117	Suidae	*Notochoerus*	molar fragment	0.9	1.5	84	5
KNM-ER 77116	Suidae	*Kolpochoerus*	molar fragment	-0.2	-2.0	76	7
KNM-ER 77105	Suidae	*Kolpochoerus*	lower m2, sub-adult	-1.8	-0.2	66	10

a. Oxygen isotope values calculated using an acid fractionation factor of 1.00799 based on Equation 4 in ref. [6].

Carbon isotope data from 17 fossil enamel analyses range from -1.8 to +1.8 ‰ (median, +1.4 ‰), equivalent to 66 to 90 %C_4 (median, 87 %C_4) resources in their diet (see also Figure 7). All taxa analyzed except for one suid have greater than 75 %C_4 resources in their diet. Oxygen isotope values range from -3.5 to +3.6 ‰ (median, +0.1 ‰). The single hippo had the most negative value and an alcelaphin bovid had the highest value. Carbon and oxygen isotope from pretreated fossil enamel are reported with respect to the VPDB scale. Sample were treated with 3% NaOCl followed by buffered acetic acid (0.1 M). Detailed descriptions of pretreatment and analytical protocols can be found in ref. [7].

Supplementary Table 2. Kruskal-Wallis comparisons between those reported here from Area 13 and those collected in upper Burgi sediments in the greater East Turkana region[a]

Taxon	Carbon test stat	Carbon df	Carbon p value	Carbon n Area 13	Carbon n UB	Oxygen test stat	Oxygen df	Oxygen p value	Oxygen n Area 13	Oxygen n UB
Aepycerotini/ Antilopini	1.994	1	0.158	1	15	0.250	1	0.617	1	5
Alcelaphini	1.028	1	0.311	4	25	0.250	1	0.617	4	15
Bovini	0.214	1	0.643	6	4	NA	NA	NA	6	0
Ceratotherium	NA	NA	NA	1	0	NA	NA	NA	1	0
Elephas	2.25	1	0.134	1	6	1.500	1	0.221	1	2
Equidae	3.858	1	0.050	3	19	0.670	1	0.413	3	8
Hippopotamus	2.823	1	0.093	1	30	1.289	1	0.256	1	25
Kolpochoerus	2.578	1	0.108	2	20	2.605	1	0.1.07	2	10
Notochoerus	2	1	0.157	1	4	2	1	0.157	1	4
Reduncini	0.179	1	0.673	2	16	0	1	1.000	2	8

a. There were few statistical differences in enamel isotopes between KNM-ER 2598 faunal specimens and other upper Burgi Member specimens. There was a significant difference in the carbon-13 enamel isotopes within family Equidae. It should be noted that sample sizes of specimens sampled in association with KNM-ER 2598 are extremely low compared to their upper Burgi counterparts.

Supplementary References Cited:

1 Copes, L. E. & Kimbel, W. H. Cranial vault thickness in primates: *Homo erectus* does not have uniquely thick vault bones. *J. Hum. Evol.* **90**, 120-134 (2016).

2 Lieberman, D. E. How and why humans grow thin skulls: experimental evidence for systemic cortical robusticity. *Am. J. Phys. Anthropol.* **101**, 217-236 (1996).

3 Laird, M. F. *et al.* The skull of *Homo naledi*. *J. Hum. Evol.* **104**, 100-123 (2017).

4 Brown, F., Gathogo, P. N., Leakey, M. G. & Raynolds, R. (Arcgis.com, 2019).

5 Feibel, C. *Paleoenvironments of the Koobi Fora Formation, Turkana Basin, Northern Kenya*, University of Utah, (1988).

6 Passey, B. H., Cerling, T. E. & Levin, N. E. Temperature dependence of oxygen isotope acid fractionation for modern and fossil tooth enamels. *Rapid Communications in Mass Spectrometry* **21**, 2853-2859 (2007).

7 Uno, K. T. *et al.* Large mammal diets and paleoecology across the Oldowan–Acheulean transition at Olduvai Gorge, Tanzania from stable isotope and tooth wear analyses. *J. Hum. Evol.* **120**, 76-91 (2018).
