# Supplementary material for: New hominin remains and revised context from the earliest Homo erectus locality in East Turkana, Kenya
Source: Nat Commun. 2021 Apr 13;12:1939. doi: 10.1038/s41467-021-22208-x (PMC8044126; doi:10.1038/s41467-021-22208-x)
Supplement: Supplementary file 3 — Description of Additional Supplementary Files [file 41467_2021_22208_MOESM3_ESM.pdf]

Description of Additional Supplementary Files

File Name: Supplementary Data 1
Description: This dataset includes a Google Earth file with source data for Figure 3
