# Supplementary material for: New hominin remains and revised context from the earliest Homo erectus locality in East Turkana, Kenya
Source: Nat Commun. 2021 Apr 13;12:1939. doi: 10.1038/s41467-021-22208-x (PMC8044126; doi:10.1038/s41467-021-22208-x)
Supplement: Supplementary file 5 — Reporting Summary [file 41467_2021_22208_MOESM5_ESM.pdf]

nature research

Corresponding author(s): Hammond, A. S.

Last updated by author(s): Mar 2, 2021

Reporting Summary

Nature Research wishes to improve the reproducibility of the work that we publish. This form provides structure for consistency and transparency in reporting. For further information on Nature Research policies, see our Editorial Policies and the Editorial Policy Checklist.

Statistics

For all statistical analyses, confirm that the following items are present in the figure legend, table legend, main text, or Methods section.

n/a	Confirmed	
☐	☒	The exact sample size (n) for each experimental group/condition, given as a discrete number and unit of measurement
☐	☒	A statement on whether measurements were taken from distinct samples or whether the same sample was measured repeatedly
☐	☒	The statistical test(s) used AND whether they are one- or two-sided<br>*Only common tests should be described solely by name; describe more complex techniques in the Methods section.*
☒	☐	A description of all covariates tested
☒	☐	A description of any assumptions or corrections, such as tests of normality and adjustment for multiple comparisons
☒	☐	A full description of the statistical parameters including central tendency (e.g. means) or other basic estimates (e.g. regression coefficient) AND variation (e.g. standard deviation) or associated estimates of uncertainty (e.g. confidence intervals)
☐	☒	For null hypothesis testing, the test statistic (e.g. F, t, r) with confidence intervals, effect sizes, degrees of freedom and P value noted<br>*Give P values as exact values whenever suitable.*
☒	☐	For Bayesian analysis, information on the choice of priors and Markov chain Monte Carlo settings
☒	☐	For hierarchical and complex designs, identification of the appropriate level for tests and full reporting of outcomes
☒	☐	Estimates of effect sizes (e.g. Cohen's d, Pearson's r), indicating how they were calculated

Our web collection on statistics for biologists contains articles on many of the points above.

Software and code

Policy information about availability of computer code

Data collection	No software was used.
Data analysis	R Studio version 1.1.463 was used for all data analysis

For manuscripts utilizing custom algorithms or software that are central to the research but not yet described in published literature, software must be made available to editors and reviewers. We strongly encourage code deposition in a community repository (e.g. GitHub). See the Nature Research guidelines for submitting code & software for further information.

Data

Policy information about availability of data

All manuscripts must include a data availability statement. This statement should provide the following information, where applicable:

- Accession codes, unique identifiers, or web links for publicly available datasets
- A list of figures that have associated raw data
- A description of any restrictions on data availability

The data supporting the findings of this study are available within the paper, its supplementary information files, or as an upload to a data-sharing repository. Datasets are provided for Figs. 3, 4, 7, and Supplementary Fig. 5. Restrictions apply to the availability of hominin scan data figured herein, but these data are available from the corresponding author with the permission of the authorizing third party (museum or individual).

Field-specific reporting

Please select the one below that is the best fit for your research. If you are not sure, read the appropriate sections before making your selection.

☐ Life sciences ☐ Behavioural & social sciences ☒ Ecological, evolutionary & environmental sciences

For a reference copy of the document with all sections, see nature.com/documents/nr-reporting-summary-flat.pdf

Ecological, evolutionary & environmental sciences study design

All studies must disclose on these points even when the disclosure is negative.

Study description	This study examines the geological and paleoecological context of hominin fossils from collection Area 13 in East Turkana, northern Kenya.
Research sample	The research sample associated with this project includes (1) fossil specimens, (2) published and new stable carbon and oxygen isotope data from fossil enamel, (3) geologic specimens.
Sampling strategy	New fossil and geologic samples were collected at the field site in East Turkana. Fossil sample sizes were dictated by the available paleontological material exposed on the surface of the study area. Isotopic sample sizes were determined based on the suitability of the collected fossils for sampling. The comparative sample for isotopic analyses was based on the available published comparative data for carbon and oxygen isotopes from East Turkana. There are no standard criteria for which to determine sample sizes for geological analyses included in this paper.
Data collection	Paleontological specimens were collected by ASH. Isotopic samples were collected by MB. Geological samples were collected by authors SM and DVP.
Timing and spatial scale	Paleontological specimens were collected and analyzed in the summers of 2017-2019 by the senior author (ASH). Isotopic samples were collected in the summer of 2019 (by author MB) and analyzed in the fall of 2019 (by author KTU). Geological samples were collected in summer 2019, and analyzed in fall 2019-spring 2020 (by authors SM and DVP).
Data exclusions	No data were excluded.
Reproducibility	This only applies to the enamel isotopic analyses. These were obtained in relation to known standards and universally accepted Standard Operating Procedures.
Randomization	Specimens were grouped into traditional taxonomic units for analyses (tribe, genus, or species).
Blinding	Blinding was not fully possible nor was it deemed necessary in this study. Author KTU was blinded to the taxonomic identification of the mammalian dental enamel for isotopic analyses. Other data were analyzed with knowledge of group membership but without expectations of what the results would be.

Did the study involve field work? ☒ Yes ☐ No

Field work, collection and transport

Field conditions	Conditions in East Turkana, Kenya, are typically hot (in excess of 35 Celsius) and dry.
Location	Samples were obtained from the fossil outcrops of East Turkana, northern Kenya.
Access & import/export	Fossil and geological samples were collected by the authority of the National Museums of Kenya (NMK) as mandated by Kenyan law. The PI (Hammond) retained permits from the Kenyan National Commission for Science, Innovation, and Technology (permits NACOSTI/P/17/46866/17343 effective July 26, 2017; NACOSTI/P/18/46866/25344 effective October 18, 2018) for field research. Excavation and Exploration license Ref NMK/GVT/2 was obtained from the Ministry of Sports, Culture, and Heritage through the NMK. Export of geological samples was conducted through a Material Transfer Agreement between the NMK, The George Washington University, and the University of the Witwatersrand, and authorized by the Kenyan Department of Mining. Export of small amounts of enamel powder for isotopic studies does not require an export permit from NMK's Parent Ministry but a Material Transfer Agreement was obtained from the NMK (loan NMK/231/42).
Disturbance	No disturbance was caused by this study (e.g., there were no excavations performed).

Reporting for specific materials, systems and methods

We require information from authors about some types of materials, experimental systems and methods used in many studies. Here, indicate whether each material, system or method listed is relevant to your study. If you are not sure if a list item applies to your research, read the appropriate section before selecting a response.

Materials & experimental systems

n/a	Involved in the study
☒	☐ Antibodies
☒	☐ Eukaryotic cell lines
☐	☒ Palaeontology and archaeology
☒	☐ Animals and other organisms
☒	☐ Human research participants
☒	☐ Clinical data
☒	☐ Dual use research of concern

Methods

n/a	Involved in the study
☒	☐ ChIP-seq
☒	☐ Flow cytometry
☒	☐ MRI-based neuroimaging

Palaeontology and Archaeology

Specimen provenance	Specimen provenance information is provided in Data Source file (Excel) and the Supplementary Data 1 (.KMZ, for Google Earth). Provenance was determined based on the established stratigraphy and paleontological collection areas in East Turkana.
Specimen deposition	National Museums of Kenya
Dating methods	We relied on published ages for tuffs. No new dating was conducted.

☒ Tick this box to confirm that the raw and calibrated dates are available in the paper or in Supplementary Information.

Ethics oversight	The Kenyan National Commission of Science, Innovation, and Technology approved all aspects of this study by issuing permits for field research to the PI (permits NACOSTI/P/17/46866/17343; NACOSTI/P/18/46866/25344). The National Museums of Kenya oversaw the field work conducted in this study.

Note that full information on the approval of the study protocol must also be provided in the manuscript.
